# Long-Term Impact of Nutritional Intervention with Increased Polyphenol Intake and Physical Activity Promotion on Oxidative and Inflammatory Profiles in Patients with Metabolic Syndrome

**DOI:** 10.3390/nu16132121

**Published:** 2024-07-03

**Authors:** Maria Magdalena Quetglas-Llabrés, Margalida Monserrat-Mesquida, Cristina Bouzas, Silvia García, David Mateos, Lucía Ugarriza, Cristina Gómez, Antoni Sureda, Josep A. Tur

**Affiliations:** 1 Research Group on Community Nutrition and Oxidative Stress, University of the Balearic Islands-IUNICS, 07122 Palma, Spainmargalida.monserrat@uib.es (M.M.-M.); pep.tur@uib.es (J.A.T.); 2 Health Research Institute of Balearic Islands (IdISBa), 07120 Palma, Spain; 3 Physiopathology of Obesity and Nutrition (CIBEROBN), Instituto de Salud Carlos III, 28029 Madrid, Spain; 4 Clinical Analysis Service, University Hospital Son Espases, 07198 Palma, Spain

**Keywords:** obesity, cardiovascular disease, intervention, oxidative stress, inflammation

## Abstract

Obesity and overweight pose significant risks to health, contributing to the prevalence of chronic conditions like type 2 diabetes mellitus (T2DM) and cardiovascular diseases (CVD). The current study aimed to assess the impact of a 6-year nutritional and lifestyle intervention on oxidative and inflammatory markers in individuals aged 55 to 75, specifically those at high risk of CVD. A study was carried out in a group of 80 participants with metabolic syndrome (MetS) residing in Mallorca, Spain, who underwent nutritional intervention based on a low-calorie Mediterranean diet (MedDiet) and promotion of physical activity. Before and after the intervention, several parameters including anthropometric data, haematological factors, blood pressure, and physical activity level were measured. Oxidative and inflammatory biomarkers in plasma were analysed. After the 6-year intervention, participants who managed to reduce their body mass index (BMI) had greater reductions in abdominal obesity, waist to heigh ratio (WHtR), diastolic blood pressure, and glucose levels, and increased high density protein cholesterol (HDL-c) compared to those who did not reduce BMI. This higher reduction in BMI was related to reduced energy intake and increased adherence to MedDiet, with greater polyphenol intake, and total physical activity (PA). Furthermore, improvements in oxidative stress and proinflammatory status were observed in participants who reduced their BMI. Significant reductions in the activity of the prooxidant enzyme, myeloperoxidase (MPO), levels of the lipid oxidation marker, malondialdehyde (MDA), and the proinflammatory chemokine, monocyte chemoattractant protein-1 (MCP-1,) were found in those who reduced their BMI. In contrast, participants who did not improve their BMI exhibited higher levels of proinflammatory markers such as MCP-1 and tumour necrosis factor α (TNFα), as well as increased activity of the antioxidant enzyme catalase (CAT). Current findings suggest that an effective way to reduce BMI is a hypocaloric MedDiet combined with tailored physical activity to improve oxidative stress and proinflammatory status, and potentially reducing the risk of CVD.

## 1. Introduction

Metabolic syndrome (MetS) comprises a cluster of risk factors that includes abdominal obesity, high triglycerides levels, hypertension, hyperglycaemia, and low high density lipoprotein cholesterol (HDL-c) levels [1]. Obesity and MetS exhibit a strong interconnection, with oxidative stress and chronic inflammation playing pivotal roles in their pathogenesis and progression [2]. Obesity increases the risk of inflammation and oxidative stress, which in turn raises the risk of many disorders involving the components of the MetS such as insulin resistance, hypertension, and hyperlipidaemia [2]. These conditions are largely caused by an imbalance between energy intake and expenditure [3]. Moreover, fast food consumption, characterize by high-calorie and low-fibre foods, and the decrease in physical activity brought on by sedentary leisure activities and the usage of automated transportation are the two key factors contributing to the emergence of this disease [3].

MetS significantly increases the risk of type 2 diabetes mellitus (T2DM) and cardiovascular disease (CVD), both of which are associated with increased oxidative stress and chronic inflammation. Its global prevalence is increasing in Western and Asian nations, particularly in developing regions undergoing rapid socio-environmental changes [4]. According to global assessments, approximately one-third of the world’s population, mainly in less economically developed areas, is affected by MetS [5]. MetS and its risk factors not only affect patient health, but are also associated with increased use of medical services and higher healthcare costs [6].

Although numerous variables contribute to the pathophysiology of MetS, multiple studies demonstrate that oxidative stress and chronic inflammation are central to the development of metabolic illnesses [7,8]. Oxidative stress, caused by an imbalance between oxidants and antioxidants, disrupts redox signalling and control, resulting in molecular and cellular damage [9]. Lipid metabolite buildup in both adipose and non-adipose tissues contribute to chronic inflammation by recruiting and activating macrophages [10]. In MetS, oxidative stress deregulates adipocytokines such as adiponectin, irisin, monocyte chemoattractant protein-1 (MCP-1), tumour necrosis factor-α (TNFα), leptin, and interleukin-6 (IL-6). Proinflammatory cytokines like TNFα, interleukin-1β (IL-1β), and IL-6, produced primarily by macrophages infiltrating adipose tissue due to obesity, are linked to insulin resistance and MetS features [7]. There is a complex interaction between obesity, chronic inflammation, oxidative stress, and the renin-angiotensin-aldosterone system (RAAS) in the development of metabolic diseases such as T2DM and CVD. Historically it has been recognized that excess body weight is closely linked to increased morbidity and mortality [11]. Adipose tissue, traditionally viewed solely as a lipid depot, is now acknowledged as an active endocrine organ that secretes inflammatory mediators and adipokines, exacerbating systemic inflammation and contributing to the development of insulin resistance and cardiovascular dysfunction [12]. These findings highlight the critical importance of weight management strategies and the implementation of targeted therapies that modulate inflammation and RAAS activity to mitigate the impact of obesity on global public health.

Several studies have shown that the risk for metabolic syndrome can be greatly reversed by reducing body weight and focusing interventions on dietary changes such as time-restricted eating, special diets such as the Mediterranean diet (MedDiet), increasing physical activity, sleep changes, or even reducing stress [2]. MedDiet is characterized by an intake consisting of greater consumption of extra virgin olive oil, whole cereal grains, fruits, vegetables, legumes, and nuts. It also entails low to moderate intake of dairy products, red meat, and red wine, alongside limited consumption of sweets and eggs. This dietary pattern has been associated with numerous health benefits [13]. MedDiet is known to provide elevated levels of phytochemicals, particularly dietary polyphenols, which offer various beneficial biological effects, including antioxidant, anti-inflammatory, immunomodulatory, antitumoral, antidiabetic, and anti-obesity activities [14]. Polyphenols, which encompass phenolic acids, flavonoids, stilbenes, and lignans, are abundant in several food sources. Flavonoids, for instance, are found in tea, fruits, soybeans, and onions, while phenolic acids are present in coffee, cereal grains, and certain fruits. Stilbenes, such as resveratrol, are prevalent in grapes and berries, and lignans are found in flax seeds, sesame seeds, and whole grains [15]. Recent studies have emphasized the diverse health benefits of dietary polyphenols. For instance, pterostilbene activates the Sirtuin 1-AMP-activated protein kinase (Sirt1-AMPK) and nuclear factor erythroid 2-related factor 2 (Nrf2) pathways in the liver, promoting fatty acid oxidation, inhibiting lipid synthesis via Sirt1–AMPK, and enhancing antioxidant defenses through Nrf2 [16]. Similarly, rutin supplementation reduces inflammatory cytokine production, improves antioxidant capacity, and enhances gut health under cold stress by modulating the toll-like receptor 4/nuclear factor-κB (TLR4/NFκβ) pathway and restoring intestinal microbiota balance [17].

The aim of the current study was to assess the effects of a 6-year nutritional and lifestyle intervention, aimed at increasing in the intake of bioactive compounds such as polyphenols and reduce sedentary behaviours, on BMI reduction and oxidative and inflammatory markers in older Spaniards with metabolic syndrome and high risk of CVD.

## 2. Materials and Methods

### 2.1. Study Design and Participants

Eighty adults from Mallorca, consisting of men (69%) aged 55–75 and women (31%) aged 60–75, were enrolled in the study. Participants were eligible if they met at least three of the following criteria for MetS: (1) abdominal obesity (waist circumference ≥ 120 cm in men and ≥80 cm in women), (2) elevated triglyceride levels (≥150 mg/dL) or receiving medication for high triglycerides, (3) low levels of high-density lipoprotein cholesterol (<40 mg/dL in men and <50 mg/dL in women), (4) high blood pressure (systolic blood pressure ≥ 130 mmHg or diastolic blood pressure ≥ 85 mmHg or receiving antihypertensive medication), and (5) elevated fasting plasma glucose (≥100 mg/dL) or receiving medication for T2DM. Additionally, participants were overweight or obese (body mass index (BMI) ≥ 27 and <40 kg/m^2^) and had no documented history of CVD. These criteria align with the updated harmonized definition provided by the International Diabetes Federation, the American Heart Association, and the National Heart, Lung, and Blood Institute [18]. Exclusion criteria encompassed the inability to provide written consent or adhere to the recommended diet or intervention visits, previous history of CVDs, active malignant cancer or history of cancer, bowel disease, liver dysfunction, and allergies to any component of the MedDiet.

**Figure 1:**
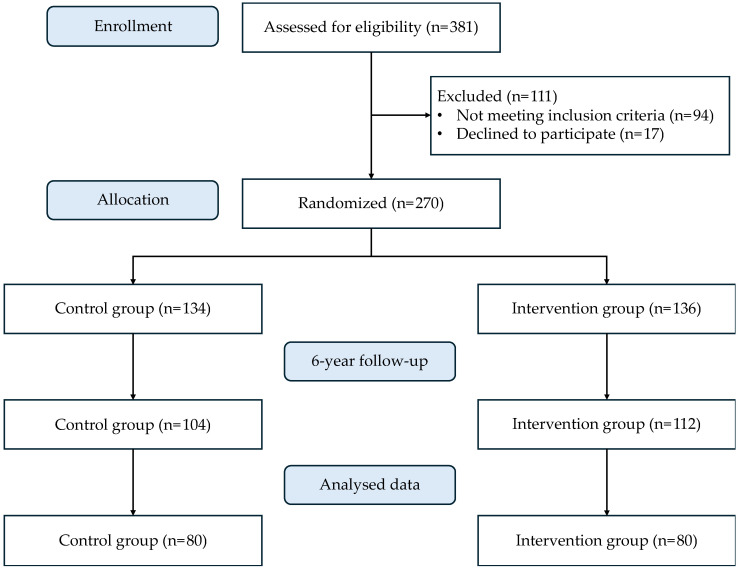
Flowchart of the study.

In this study, data from a randomised trial’s baseline and 6-year parallel groups were prospectively cohort-analysed. The objective was to evaluate the combined impact of dietary changes and physical activity. Participants were randomly assigned to one of two interventions: an intensive program involving a low-calorie MedDiet, promotion of physical activity, and behavioural therapy focused on weight loss, or a less intensive program involving an energy unrestricted MedDiet alongside standard cardiovascular prevention care. There were 381 patients examined for eligibility; 94 did not match the requirements, and 17 declined to participate. Finally, 270 patients were assigned in a 1:1 ratio to one of the two therapeutic groups for six years. After a 6-year follow-up, a sub-sample 80 subjects were selected to be examined for oxidative and inflammatory data (Figure 1). Patients were selected according to the change in their BMI throughout the 6 years of intervention regardless of the experimental group. Thus, of the total number of patients, 40 (with 20 of each gender) who showed a significant reduction in their BMI were selected (classified as responders), while the other 40 did not present an improvement after 6 years (classified as non-responders).

Every participant received detailed information regarding the study’s objectives and potential implications, and each provided written consent. The study adhered to the ethical principles outlined in the Declaration of Helsinki, and all protocols were approved by the Ethics Committee of Research of Balearic Islands (reference CEIC-IB/2251/14PI).

### 2.2. Anthropometric Measurements

At baseline and at 6-year follow-up anthropometric variables were measured twice by trained personnel. Height in centimetres was measured using a wall-mounted stadiometer with a mobile anthropometer (Seca 214, SECA Deutschland, Hamburg, Germany) to the nearest millimetre, following the Frankfort Horizontal Plane position. Weight in kilograms was determined utilizing a Segmental Body Composition Analyzer according to the manufacturer’s guidelines (Tanita BC-418, Tanita, Tokyo, Japan). Participants were weighed while wearing light clothing and no shoes, with a deduction of 0.6 kg accounted for clothing weight. BMI was computed as weight (kg) divided by height squared (m^2^). An anthropometric tape was used to measure the waist circumference in centimetres. The waist measurement, which indicates abdominal obesity, was collected at the halfway between the iliac crest and the final rib. Waist-to-height ratio (WHtR) was calculated by dividing waist circumference (cm) by height (cm). Blood pressure was recorded using a validated semi-automatic oscillometer (Omron HEM, 705CP, Hoofddrop, The Netherlands) while participants were seated for a period of 3–5 min of quiet rest; the average of 2–3 blood pressure measurements was taken.

### 2.3. Blood Collection and Analysis

Blood samples were obtained from the antecubital vein following a 12-h overnight fast using suitable vacutainers, both with and without ethylenediaminetetraacetic acid (EDTA) as an anticoagulant, to collect plasma and serum, respectively. Standard enzymatic methods were employed to conduct general blood biochemical analyses on fasting serum at the clinical laboratory of Son Espases Hospital (Palma, Spain). Serum levels of glucose, HbA1c, triglycerides, HDL-cholesterol, LDL-cholesterol, and total cholesterol were measured. Haematological parameters and a complete blood count were assessed using an automatic flow cytometer analyser Technicon H2 VCS system (Bayer, Leverkusen, Germany).

One fresh EDTA-containing blood tube underwent centrifugation at 1700× *g* for 15 min at 4 °C. Another aliquot of EDTA-containing blood was utilized to isolate neutrophils using a previously described technique [19]. Briefly, 6 mL of blood was layered onto 4 mL of Ficoll-Paque PLUS reagent and centrifuged at 900× *g* for 30 min at 4 °C. The cells at the bottom phase were incubated at 4 °C for 30 min with 0.15 M ammonium chloride (NH_4_Cl) to lyse erythrocytes. The suspension was then centrifuged at 750× *g* for 15 min at 4 °C, and the supernatant was discarded. The neutrophil phase at the bottom was initially washed with NH_4_Cl and subsequently with PBS at pH 7.4.

Samples were kept at −80 °C until they were analysed in the laboratory.

### 2.4. Dietary Assessment, Physical Activity and Sedentarism

A 143-item food frequency questionnaire (FFQ), administered by registered dieticians and validated in Spain, was employed to evaluate the dietary patterns of participants over the past 12 months [20]. Each item in the questionnaire included a standard serving size, and participants indicated their consumption frequency on a scale of nine categories ranging from “never or hardly ever” to “≥6 times/day”. To determine energy and nutrient intake, the frequency of consumption for each item was multiplied by the nutrient composition of the specified serving size. This computation was conducted using a computer program that integrates data from food composition tables [21]. The frequency data were converted into daily intake. For all foods listed in the FFQ, the average quantity consumed (in grams), total energy intake, and macro- and micronutrient levels were estimated. Additionally, the total intake of nutrients and micronutrients from dietary supplements, as reported by participants, was considered.

The 17-item questionnaire was used to assess adherence to an energy-restricted Mediterranean Diet (erMedDiet) [22]. More total scores indicate more adherence; the range of scoring is 0 to 17.

Dietary consumption of aglycone polyphenols was assessed using the European Phenol Explorer database [23]. This process standardises data from various analytical methodologies, allowing for cross-study comparisons [24]. Polyphenol intake was computed in mg/day using food consumption data from the FFQ and aglycone polyphenol content from the Phenol Explorer database. No retention factor was used. The Phenol Explorer data includes polyphenol concentrations from both chromatography and hydrolysis-based techniques.

The rate of energy expenditure indicated by existing knowledge was taken into consideration when estimating the degree of physical activity using metabolic equivalents (METs) [25]. Every participant detailed the volume of tasks finished in minutes or weeks. Sedentary behaviours were assessed using the validated Spanish version of the Nurses’ Health Study questionnaire [26], which included a series of open-ended questions about the average daily time spent over the previous year sitting while using a computer, watching TV, travelling by car, bus, or tube, driving for work or pleasure, being employed, retiring, and overall sitting. Twelve categories with sitting times ranging from 0 to ≥9 h per day for the matching activity were included in the answers.

### 2.5. Enzymatic Activities Determination

The Shimadzu UV-2011 spectrophotometer (Shimadzu Corporation, Kyoto, Japan) was used to quantify catalase (CAT) and superoxide dismutase (SOD), myeloperoxidase (MPO) enzymes in plasma at 37 °C. The breakdown of H_2_O_2_ at 240 nm was used to measure CAT activity in plasma using Aebi’s spectrophotometric approach [27]. SOD activity in plasma was evaluated using a modification of McCord and Fridovish’s approach following cytochrome C oxidation at 550 nm [28]. MPO activity in plasma was evaluated by guaiacol oxidation and measuring the resulting tetraguaiacol compound at 470 nm [29].

### 2.6. Malondialdehyde Assay

Malondialdehyde (MDA) was measured using the specific colorimetric assay kit (Sigma-Aldrich Merck^®^, St. Louis, MO, USA) following the manufacturer’s instructions. Briefly, plasma samples or standards were introduced in glass tubes containing *n*-methyl-2-phenylindole in methanol: acetonitrile mixture (1:3). 75 μL HCl (12N) were added, and samples were incubated for 60 min at 45 °C. After that, the absorbance was measured at 586 nm (Epoch, BioTek^®^ Instruments GmbH, Bad Friedrichshall, Germany) and the MDA concentration was calculated with a standard curve of known concentrations.

### 2.7. Polyphenols Assay

The total polyphenol content of plasma samples was evaluated using the Folin-Ciocalteu technique in the supernatants of deproteinized samples with cold acetone (1:1.2) and L-tyrosine as a reference. Once the reaction began, it was allowed to stand for 1.5 h before the absorbance was measured at 760 nm using a microplate spectrophotometer (Epoch, BioTek^®^ Instruments GmbH, Bad Friedrichshall, Germany).

### 2.8. Stimulated Neutrophils ROS Production

The production of radical oxygen species (ROS) neutrophils was assessed following activation with lipopolysaccharide (LPS) (100 μg/mL phosphate-buffered saline—PBS) from Escherichia coli (Sigma-Aldrich, St. Louis, MO, USA). A 96-well microplate received 50 μL of cell suspension containing approximately 6 × 10^5^ cells, followed by the addition of 50 μL of LPS to the respective wells. Subsequently, 2,7-dichlorofluorescein-diacetate (DCFH-DA, 61.6 μM in Hanks’ Balanced Salts Medium) was added to all wells as an indicator. Fluorescence (Ex, 480 nm; Em, 530 nm) was measured at 37 °C for 1 h using a FLx800 Microplate Fluorescence Reader (Bio-tek Instruments, Inc., Winuschi, VT, USA) with punctual ultraviolet light exposures, and emission readings were recorded every minute. ROS concentration was determined by measuring the fluorescence of a standard curve of known ROS concentration after its reaction with DCFH-DA under the same conditions as the samples.

### 2.9. Immunoassay Kits

Monocyte chemotactic protein-1 (MCP-1) and interleukin 1β (IL-1β) plasma levels were measured using ELISA kits following the supplies guidelines for use (RayBiotech, Peachtree Corners, GA, USA). The overall intra-assay coefficient of variation was calculated to be <10% and the inter-assay coefficient of variation was <12% for both. Tumour necrosis factor α (TNFα) plasma levels were determined using Human TNFα ELISA kits (Diaclone, Besancon Cedex, France). The overall intra-assay coefficient of variation for TNFα was calculated to be 3.2%, while the overall inter-assay coefficient of variation for TNFα was calculated to be 10.9%.

Immunoassay kits were utilized in accordance with the manufacturer’s instructions for comprehensive and accurate results and read at 450 nm (Epoch, BioTek^®^ Instruments GmbH, Bad Friedrichshall, Germany).

### 2.10. Statistics

Statistical analysis was carried out with the Statistical Package for Social Sciences (SPSS v.29, IBM Software Group, Chicago, IL, USA). Data are shown as mean ± standard deviation (SD), considering *p* < 0.05 as statistically significant. The Kolmogorov–Smirnov test was used to assess the normality of the data. A two-way analysis of covariance (ANCOVA) after adjustment for age and gender was used to check the significance of the data. A Bonferroni post hoc test was carried out when significant differences were found between groups.

## 3. Results

**Figure 2:**
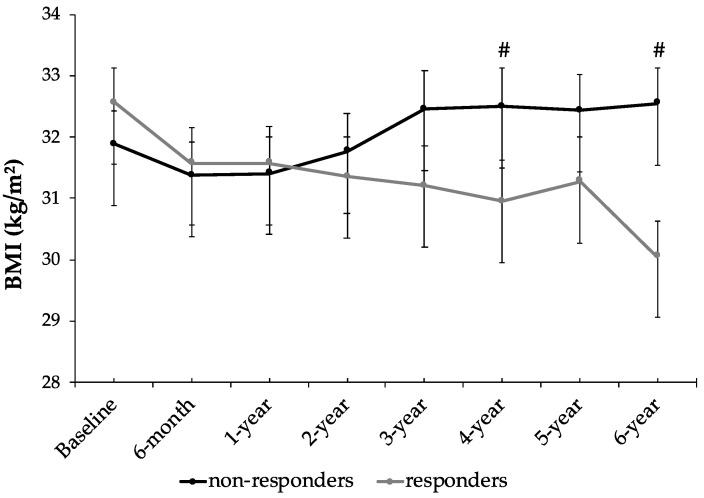
Body mass index (BMI) in MetS participants during the 6 years of lifestyle intervention, comparing responders with non-responders. Results are presented as mean ± standard error of the mean (SEM). # Differences in means between groups (responders and non-responders) at the same time by Student’s *t*-test for impaired data. Data points are significant when *p* < 0.05.

At the beginning of the study, no significant differences were found between mean ages of the participants in the responder group (63.6 ± 5.6 years) and non-responder group (63.2 ± 4.9 years). Figure 2 shows the changes in BMI during the 6 years of lifestyle intervention, comparing those with metabolic syndrome who achieved a reduction in their BMI (responders) with those who did not achieve a reduction in their BMI after the 6-year intervention (non-responders). Throughout the study, there is a progressive reduction in BMI of responders until 4 years of intervention. After 5 years of intervention, the responders slightly increased their BMI, corresponding to data collected after the COVID-19 pandemic, where follow-ups were complicated by confinement, and after 6 years they decreased again. On the other hand, the non-responder participants, while initially managing to reduce their BMI and maintain it during the first year, experience a progressive increase during the second and third years before stabilizing in the subsequent years. At the end of the 6-year follow up, the group of responders presented significantly lower BMI than the non-responder group (*p* < 0.05).

Table 1 shows the anthropometric, clinical, and haematological parameters of MetS participants, comparing responders who achieved a reduction in their BMI after 6 years and non-responders who did not, both at baseline and after the 6-year intervention. On one hand, participants who did not achieved a reduction in their BMI (non-responders) after a lifestyle intervention increased their systolic blood pressure, HbA1c levels and monocytes count. On the other hand, the responder group showed a significant reduction in their BMI, which was significantly lower than that of the non-responders. It should be noted that at baseline, responders presented higher abdominal obesity, high glucose levels and low HDL-c levels compared to non-responders’ participants. After the 6-year intervention responders participants reduce significantly their WtHR and abdominal obesity. Moreover, they reduce their diastolic blood pressure, glucose levels and increase their HDL-c levels.

In Table 2, the dietary assessments and physical activity of participants are presented at baseline and after 6 years of a lifestyle intervention, categorized by those who achieved a reduction in their BMI (responders) and those who did not (non-responders). There were no significant differences in these parameters between the two groups at baseline, except for folic acid intake, which was significantly lower in participants who did not respond. This indicates that the two groups were comparable at the start of the study, except for their folic acid intake levels. Regarding dietary assessment, both groups significantly increased their adherence to the MedDiet, omega-3 fatty acids (ω-3 FA), fibre, and folic acid consumption, and reduced their glycaemic index. Moreover, only responders significantly reduced their energy intake, glycaemic load, carbohydrates, and SFA intake, while also increasing their consumption of vitamin C and polyphenols, influenced by a dietary pattern based on vegetables and fruits. Specifically, responders consumed significantly more flavonoids than non-responders after the 6-year intervention. Both groups increased their phenolic acid intake; however, non-responders significantly increased their lignan and stilbene consumption. On the other hand, participants who achieved a reduction in their BMI significantly reduced their sedentary time and increased their total physical activity. Despite observing a trend towards increasing light and moderate physical activity, no significant differences were observed between light, moderate, and vigorous physical activity practices.

Table 3 presents the indicators of oxidative stress in plasma and neutrophils of individuals at the start of the study and after 6 years of a lifestyle intervention, distinguishing between those who achieved a reduction in BMI (responders) and those who did not (non-responders). After 6 years, participants who did not reduce their BMI showed an increase in plasma CAT activity, as antioxidant enzyme, while SOD activity did not change between the groups. Participants who responded and reduced their BMI also showed reductions in MPO plasma activity, as prooxidant enzyme, MDA plasma levels, formed as secondary products during peroxidation of polyunsaturated fatty acids and used as lipid oxidation marker, and ROS production in neutrophils stimulated with LPS.

**Figure 3:**
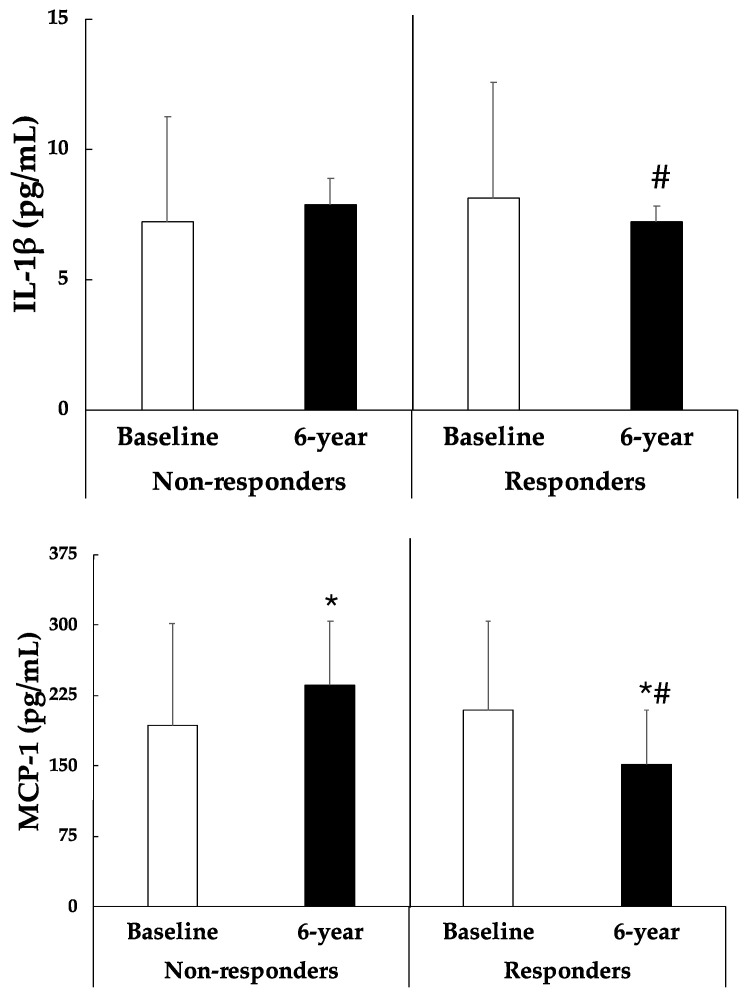

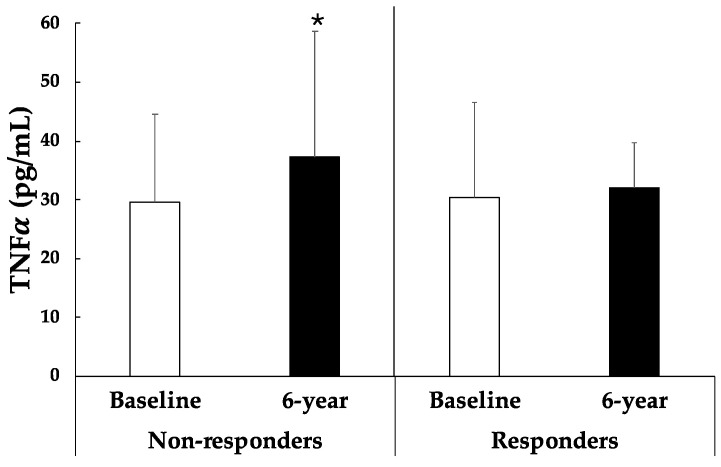
Interleukin 1β (IL-1β), monocyte chemotactic protein-1 (MCP-1) and tumour necrosis factor α (TNFα) in MetS participants during the 6 years of lifestyle intervention, comparing responders with non-responders. Results are presented as mean ± SD (standard deviation). Two-way analysis of co-variance (ANCOVA) after adjustments by age and sex. * Difference in means between participants in time (baseline and 6-year). # Difference in means between groups (responders and non-responders). Data points are significant when *p* < 0.05.

Figure 3 illustrates pro-inflammatory cytokines levels of participants at baseline and after 6-year of a lifestyle intervention regarding those who achieved a reduction in BMI (responders) and those who did not (non-responders). After the lifestyle intervention, participants who responded present significantly lower levels of IL-1β and MCP-1 than those who did not. In fact, MCP-1 significantly increased in non-responders while it decreased in responders after 6 years. TNFα levels were significantly increased in non-responders whereas no changes were observed in responders after 6 years.

## 4. Discussion

The main findings of the present study are the improvement of the oxidative and proinflammatory prolife in those participants with MetS who improved their BMI after 6 years of lifestyle intervention based in an erMedDiet and physical activity promotion. During the follow-up period of 6 years, anthropometric measurements were taken, and this study, was focused on the evolution of BMI. The results revealed that the two groups follow a differentiated response pattern, since while the group of responders was able to maintain the reduction in BMI achieved throughout the first year and even continue to decrease more gradually, the group of non-responders, after the initial year it recovered the initial values and even exceeded them slightly from the third year onwards. Preventing weight regain after successful weight loss is a major challenge in obesity management. In fact, it is well described that many individuals regain weight over months or years despite significant effort mainly due to the obesogenic environment to which we are subjected, making it difficult to maintain healthy habits in the long term [30,31]. Effective strategies are urgently needed to sustain weight loss and its health benefits, including various approaches beyond dietary changes. Obesity treatment success should be evaluated not just by weight loss, but also by lasting improvements in diet quality and physical activity. These long-term lifestyle changes can enhance health even without substantial weight loss.

After 6 years of intervention, participants who reduced their BMI, also reduced significantly their WHtR, hip circumference and abdominal obesity, being these values significantly lower than those participants who did not achieve reduce their BMI at 6 years. While non-responders, increased systolic blood pressure within the normal range, yet slightly higher, suggesting a potential link between non-response and adverse cardiovascular outcomes. These results are in accordance with previous studies reporting that a significant weight loss is related with a reduction in waist circumference, which in turn, leads to improvements in various components of the MetS, including blood pressure, lipid profiles, and glycaemic measures, regardless of sex [32,33]. However, even though responders reduced significantly their diastolic pressure after the intervention, no change was observed in their systolic pressure, as suggested previous bibliography [34]. Our results are consistent with findings reporting that insulin resistance during obesity is driven by an inflammatory response caused by the infiltration of adipose tissue by monocytes and other leukocytes, leading to disrupted insulin signalling and hyperglycaemia. Activated immune cells release cytokines such as TNFα and IL-1β, which activate a series of intracellular signalling pathways, altering insulin signalling and inducing insulin resistance. This inflammation is also directly linked to hypertension and hyperlipidaemia [35]. Participants who worsened their BMI also showed an increase in monocyte levels, highlighting the role of these cells in obesity-induced inflammation.

In general, all participants increased their adherence to the MedDiet after 6-year intervention, with significant differences in both groups. However, our findings indicate that only participants who achieved a reduction in BMI also demonstrated greater adherence to the MedDiet along with a significant reduction in caloric intake. This aligns with a previous study that reported significant changes in body weight, BMI, and body composition after an 8-month intervention with erMedDiet [36]. Both studies highlight that the combination of a MedDiet and caloric reduction were more effective for weight loss and BMI reduction compared to the MedDiet alone. Moreover, our results support the notion that greater adherence to the MedDiet was inversely associated with the risk of overweight and/or obesity [37]. However, unlike the previous study, our long-term results emphasize that sustained BMI reduction is associated with continuous dietary behaviour changes, highlighting the importance of ongoing adherence to a balanced diet and caloric control for achieving significant improvements in body composition and metabolic parameters.

A review analysing all the available literature from 2019 and 2020 focussed on carbohydrates and weight loss concluded that there are no significant differences in weight recovery despite variations in carbohydrate, protein, and fibre intake [38]. However, there are indications that certain subgroups of people, such as those with higher fasting glucose levels, may benefit more from diets with a reduced glycaemic load. Diet adherence and individual characteristics, such as genetics and microbiome, may also influence the effectiveness of these diets [38]. The results of our study suggest a significant association between the modification of carbohydrate intake, glycaemic load, and improvement in BMI among patients with MetS undergoing a six-year intervention. In this sense, it is widely described that long-term ingestion of diets with high glycaemic load and glycaemic index, characterized by high amounts of refined carbohydrates and low-fibre foods, over time is associated with a greater risk of obesity and cardiometabolic diseases [39]. In the present study, we observed that those patients who experienced an improvement in their BMI post-intervention showed a significant reduction in their carbohydrate intake and an enhancement in their glycaemic load compared to those who did not show BMI improvement. Although both groups improved their glycaemic index, the group that experienced a reduction in BMI demonstrated a greater tendency towards decreased glycaemic load. These findings suggest that the quality and quantity of carbohydrates consumed may have a significant impact on weight control and metabolic health in patients with MetS. The reduction in carbohydrate intake and improvement in glycaemic load could be considered effective strategies in the long-term management of this clinical condition.

In both groups, participants significantly increased their intake of ω-3 fatty acids, folic acid, and fibre. These results are in accordance with previous studies indicating that a characteristic of the MedDiet is the greater consumption of antioxidants such as selenium, vitamins C and E, dietary fibre, and few simple sugars. This could explain that increased adherence to the MedDiet is associated with significantly higher average consumption of ω-9 fatty acids, dietary fibre, and lower consumption of SFA, differing from what we found in the standard diet group [40]. Our study evidenced that participants who reduced their BMI also significantly decreased their intake of SFA and increased their vitamin C intake. Vitamin C, with its antioxidant and anti-inflammatory properties, enhances fatty acid oxidation and may reduce adiposity, suggesting its role in improving metabolic health. These findings highlight the potential benefits of dietary modifications, such as reducing SFA and increasing vitamin C intake, for managing BMI and MetS [41].

A recent meta-analysis supported that higher polyphenol intake can lower the risk of MetS [42]. In the present study, only the participants who reduced their BMI had a substantial rise in their overall consumption of polyphenols. In particular, during the 6-year intervention, responders significantly ingested more flavonoids than non-responders. Flavonoids, bioactive polyphenolic compounds derived from plants, have been observed to inhibit the NF-κB p65 and MAPK pathways, leading to reduced inflammation, and can enhance endothelial function by activating vascular Akt and eNOS. Incorporating flavonoids into one’s diet can contribute to improved vascular health and a decreased risk of developing hypertension and cardiovascular disease [43]. A recent systematic review, which included eighteen randomized trials and eleven crossover trials, demonstrated that flavonoids, when used alone as supplements in patients with MetS and related pathologies, can significantly modify several metabolic parameters and consequently reduce the risk of diseases associated with MetS, except for body weight and BMI [44]. However, the duration of these studies ranged from 2 to 12 weeks, suggesting that prolonged intake of flavonoids over a longer period could be beneficial and potentially related to weight loss. In fact, the randomized PREDIMED study confirmed that a long-term polyphenol-rich diet contributes to body weight loss in an elderly population [45]. While both groups consumed more phenolic acids, non-responders consumed considerably more lignans and stilbenes. Fruits and vegetables are characterized mainly by the presence of free phenolic acids, whereas grains and derivatives contain bound phenolic acids. This difference in phenolic acid forms and their sources could suggest variations in dietary patterns between both groups. Additionally, the higher intake of lignans and stilbenes in non-responders might indicate a preference for foods rich in these compounds, such as flaxseeds and certain berries [15], which may not have the same impact on BMI and metabolic parameters as the flavonoid-rich foods consumed by responders.

Participants who successfully reduced their BMI also significantly decreased their sedentary time and increased their overall physical activity, with a trend towards more light and moderate activities and no changes in vigorous activity. This aligns with other studies on older adults, who typically engage more in light and moderate activities due to physical limitations. A systematic meta-analysis found that the MedDiet is associated with greater weight loss, especially when combined with energy restriction and increased physical activity [46]. Another meta-analysis showed that adults with type 2 diabetes following an energy-restricted MedDiet with 175 min of weekly physical activity was one of the few interventions achieving the recommended 5% weight loss at 12 months [47]. Regular exercise can help reduce weight, lower blood pressure, and improve lipid disorders, and it is particularly effective in combating insulin resistance and lowering the prevalence and incidence of MetS [48].

Oxidative stress is characterised by an imbalance between oxidants and antioxidants, wherein the body experiences tissue injury due to lipid peroxidation, DNA damage, and protein denaturation, playing a role in the pathogenesis of MetS in various conditions [9]. Participants who achieved a reduction in their BMI after 6 years showed a decrease in MPO plasma activity, ROS production by neutrophils stimulated by LPS, and MDA plasma levels. In contrast, non-responders increased their CAT plasma activity. The results obtained in the present study are in line with previous studies. Plasma CAT activity, as an antioxidant enzyme protective against oxidative damage, has been reported to be higher in obese people compared to those of normal weight [49]. High CAT activity in obese individuals is suggestive of the presence of oxidative stress, and it is believed that this increased activity may be a compensatory mechanism to overcome the increased hydrogen peroxide content, thus mitigating potential oxidative damage to cells and tissues. Plasma MPO levels are inversely linked to obesity measures like weight, waist circumference, BMI, and insulin resistance [50]. In our study, participants who reduced their BMI not only showed a decrease in their MPO activity compared to their initial values, but also had lower MPO activity after 6 years compared to the other group. MPO presence suggests increased oxidative stress in obesity. Inflammation prompts leukocytes to produce MPO, which generates ROS crucial for microbial defence [51]. The levels of MDA and ROS in plasma were measured in this study in order to assess oxidative damage. MDA plasma levels, product from the peroxidation of polyunsaturated fatty acids and an indicator of lipid damage due to oxidation, exhibited a significant decrease in individuals who decreased their BMI over a 6-year follow-up period compared to their initial values. These results corroborate earlier studies that heightened MDA levels may result from diminished activity of antioxidant enzymes, a common occurrence in inflammatory conditions [52]. The enhancement of antioxidant defence mechanisms post-nutritional intervention likely contributed to the notable reduction in MDA levels. Additionally, this decline could be influenced by the increased presence of circulating polyphenols with antioxidant properties, linked to better adherence to the MedDiet [53]. Furthermore, a balanced diet with moderate caloric restriction, including legume servings four days a week, enhanced the improvement in oxidative stress associated with weight loss by reducing MDA levels [54]. In our recent study, we have elucidated significant findings regarding the impact of a low-calorie MedDiet intervention on the production of ROS by neutrophils stimulated with LPS over a six-year period. Comparing these results with our previous study, conducted over a two-year duration, we observed a notable decrease in ROS generation following the extended intervention [54]. Previous study suggests that an increased ROS secretion into peripheral blood from accumulated fat in obesity is also involved in induction of insulin resistance in skeletal muscle and adipose tissue, impaired insulin secretion by β cells, and pathogenesis of various vascular diseases such as atherosclerosis and hypertension. [55].

While polyphenols are well-known for their powerful antioxidant properties [14], our study did not detect differences in plasma polyphenol values between different time points and intervention groups. This observation may be attributed to the timing of sample collection, as plasma polyphenol levels typically peak 1 to 2 h after ingestion and are affected by factors such as fasting status [56]. In our study, samples were obtained after an overnight fast, potentially masking acute changes in polyphenol levels following dietary intervention

Regarding inflammatory biomarkers, the present findings reveal that non-responders experienced a significant increase in their plasma levels of MCP-1 and TNFα after the 6-year intervention, indicating a heightened proinflammatory status. In contrast, responders exhibited a significant decrease in their plasma levels of IL-1β and MCP-1 following the intervention, suggesting a reduction in inflammation. IL-1β serves as a potent proinflammatory cytokine, primarily generated by inflamed adipose tissue in humans, as well as immune cells in instances of obesity. Its presence not only exacerbates insulin signalling impairment but also elevates lipolysis [57]. Similarly, MCP-1, primarily secreted by vascular cells and within visceral adipose tissue, prompts macrophage infiltration and fosters insulin resistance [58]. Various research studies have highlighted a decline in these cytokines following weight loss induced by dietary changes, consequently ameliorating cardiometabolic risk factors [59,60]. TNFα is a cytokine widely utilized as an indicator of the pro-inflammatory state characterizing obesity and contributing to insulin resistance. While a 12-week intervention involving caloric reduction has been shown to improve TNFα levels [60], our study found that responders did not exhibit significant changes in their TNFα values compared to their initial levels. However, an increase in TNFα levels was observed in participants who did not improve their BMI after 6 years of intervention. This finding aligns with evidence indicating that individuals with higher levels of obesity tend to have elevated TNFα levels [61].

The primary strength of the study lies in the significant improvement observed in oxidative stress and proinflammatory biomarkers among participants who successfully reduced their BMI. This finding suggests potential significance for the clinical management of metabolic syndrome and could help mitigate the severity of other cardiovascular risk factors. Additionally, the presented results indicate the prolonged nature of the intervention, spanning a period of 6 years. The observed changes in oxidative stress and proinflammatory biomarkers over this timeframe provide valuable insights into the sustained benefits of the intervention. However, a limitation of this research is its relatively small sample size, which precludes certain analyses such as gender stratification. The fact that age and sex were accounted for in the statistical analysis may bolster the findings. Nevertheless, despite this limitation, the sample size was adequate for detecting differences in biomarker levels between intervention groups. Another potential limitation involves inter-observer variability in anthropometric measurements. To address this concern, meticulous personnel training was conducted to ensure measurement accuracy. Overall, while the study has notable strengths, these limitations should be considered when interpreting the results. Finally, a limitation of this study is that it only investigated the oxidative and proinflammatory status using human plasma samples. Future research could benefit from including cellular tests and signal pathway analyses, although obtaining the necessary biopsies or other sample types poses significant challenges.

## 5. Conclusions

The main conclusion of the present study is that a six-year lifestyle intervention based on a low-calorie Mediterranean diet and increased physical activity leads to significant improvements in the oxidative and proinflammatory profile among individuals with metabolic syndrome who successfully reduce their body mass index (BMI). Participants who achieve BMI reduction show improvements in anthropometric and biochemical parameters, including reductions in oxidative stress and inflammation. In contrast, those who fail to reduce their BMI show an increase in antioxidant activity and proinflammatory levels. These findings highlight the effectiveness of long-term lifestyle interventions in improving metabolic health and reducing cardiovascular risk factors in individuals with metabolic syndrome.

## Figures and Tables

**Table 1:** Anthropometric, clinical and haematological parameters of MetS participants comparing responders and non-responders at baseline and after the 6-year intervention.

	Non-Responders (*n* = 40)		Responders (*n* = 40)		
	Baseline	6-Year	Baseline	6-Year	*p*-Value
Anthropometry parameters					
Weight (kg)	86.5 ± 12.0	87.7 ± 11.9 *	90.4 ± 13.9	83.6 ± 13.9 *	<0.001
BMI (kg/m^2^)	31.9 ± 3.5	32.5 ± 3.6 *	32.6 ± 3.5	30.1 ± 3.6 *#	<0.001
WHtR	0.650 ± 0.055	0.656 ± 0.057	0.669 ± 0.041	0.629 ± 0.039 *#	<0.001
Abdominal obesity (cm)	107.1 ± 9.8	107.8 ± 10.0	112.5 ± 7.1 #	105.5 ± 8.0 *	<0.001
Clinical parameters					
Systolic blood pressure (mmHg)	139.2 ± 14.1	148.4 ± 16.4 *	144.3 ± 17.5	146.8 ± 15.7	0.280
Diastolic blood pressure (mmHg)	82.9 ± 9.9	85.0 ± 10.3	83.5 ± 12.6	77.3 ± 9.4 *#	0.010
Glucose (mg/dL)	109.8 ± 20.6	113.1 ± 31.0	124.9 ± 35.2 #	116.0 ± 24.7 *	0.037
HbA1c (%)	6.03 ± 0.77	6.28 ± 0.93 *	6.37 ± 1.01	6.26 ± 0.79	0.013
Triglycerides (mg/dL)	136.9 ± 65.6	124.2 ± 47.1	160.1 ± 63.4	148.9 ± 90.6	0.884
HDL-cholesterol (mg/dL)	45.4 ± 9.2	47.3 ± 14.5	39.9 ± 9.0 #	45.3 ± 11.9 *	0.105
LDL-cholesterol (mg/dL)	115.5 ± 30.1	104.1 ± 33.6	101.1 ± 34.8	89.9 ± 29.4	0.888
Cholesterol total (mg/dL)	184.0 ± 33.0	175.9 ± 37.9	171.9 ± 37.0	163.8 ± 34.0	0.871
Haematological parameters					
Haematocrit (%)	43.2 ± 4.1	43.1 ± 5.9	43.6 ± 3.9	43.0 ± 6.2	0.825
Erythrocytes (10^6^/mm^3^)	4.84 ± 0.47	4.77 ± 0.56	4.91 ± 0.50	4.82 ± 0.54	0.989
Leukocytes (10^6^/mm^3^)	7.07 ± 1.88	7.10 ± 1.48	7.04 ± 1.54	7.01 ± 1.68	0.814
Neutrophils (10^6^/mm^3^)	5.09 ± 7.66	3.85 ± 1.16	4.71 ± 4.63	4.10 ± 1.43	0.723
Lymphocytes (10^6^/mm^3^)	3.00 ± 4.11	2.28 ± 0.68	2.21 ± 0.61	2.03 ± 0.71	0.425
Monocytes (10^6^/mm^3^)	0.602 ± 0.204	0.673 ± 0.184 *	0.629 ± 0.157	0.636 ± 0.157	0.094
Eosinophils (10^6^/mm^3^)	0.231 ± 0.146	0.254 ± 0.180	0.275 ± 0.397	0.196 ± 0.109	0.148
Basophils (10^6^/mm^3^)	0.066 ± 0.143	0.170 ± 0.734	0.042 ± 0.022	0.055 ± 0.116	0.476

Abbreviations: BMI, body mass index; WHtR, waist to heigh ratio; HbA1c, glycated haemoglobin A1c; HDL, high density lipoprotein; LDL, low density lipoprotein. Results are presented as mean ± SD (standard deviation). Two-way analysis of co-variance (ANCOVA) after adjustments by age and sex. * Difference in means between participants in time (baseline and 6-year). # Difference in means between groups (responders and non-responders). Data points are significant when *p* < 0.05.

**Table 2:** Dietary assessment and physical activity of MetS participants comparing responders and non-responders at baseline and after the 6-year intervention.

	Non-Responders (*n* = 40)		Responders (*n* = 40)		
	Baseline	6-Year	Baseline	6-Year	*p*-Value
Dietary assessment					
Adherence erMedDiet (17-items)	7.00 ± 1.88	10.7 ± 2.8 *	7.95 ± 2.53	11.2 ± 3.1 *	0.432
Energy intake (kcal/day)	2461 ± 810	2374 ± 496	2676 ± 811	2368 ± 491 *	0.256
Glycaemic load	141.7 ± 61.6	122.3 ± 41.0	164.4 ± 71.3	127.4 ± 36.0 *	0.398
Glycaemic index	56.8 ± 3.8	53.1 ± 5.0 *	56.8 ± 5.5	53.8 ± 4.8 *	0.716
Macronutrients					
Carbohydrates (g/day)	245.7 ± 96.0	226.8 ± 64.3	285.3 ± 109.8	234.7 ± 54.6 *	0.322
Proteins (g/day)	97.1 ± 30.1	95.7 ± 15.6	100.7 ± 30.9	99.8 ± 21.6	0.856
Lipids (g/day)	110.9 ± 45.5	114.5 ± 27.8	114.0 ± 36.6	105.8 ± 25.0	0.346
Micronutrients					
MUFAs (g/day)	56.6 ± 23.8	63.9 ± 17.1	56.9 ± 17.2	60.9 ± 16.0	0.677
PUFAs (g/day)	18.2 ± 9.41	21.1 ± 5.36	20.5 ± 7.32	18.9 ± 3.8	0.095
SFA (g/day)	29.0 ± 12.7	26.0 ± 7.74	31.8 ± 15.1	25.4 ± 7.60 *	0.505
Trans FA (g/day)	0.717 ± 0.473	0.563 ± 0.294	0.802 ± 0.692	0.578 ± 0.313	0.882
ω-6 FA (g/day)	13.9 ± 7.0	16.4 ± 4.83	14.9 ± 6.83	13.9 ± 3.70 #	0.092
ω-3 FA (g/day)	1.96 ± 0.61	2.62 ± 0.80 *	2.23 ± 0.92	2.56 ± 0.94 *	0.196
Cholesterol (mg/day)	416.0 ± 137.9	351.6 ± 93.7	409.5 ± 217.0	382.8 ± 124.3	0.441
Folic acid (µg/day)	290.5 ± 76.2	399.6 ± 113.5 *	353.6 ± 117.7 #	419.4 ± 114.7 *	0.267
Fibre (g/day)	24.5 ± 9.74	33.9 ± 9.39 *	30.7 ± 11.0 #	36.0 ± 10.5 *	0.255
Vitamin A (µg/day)	1090 ± 484	1276 ± 475	1234 ± 692	1391 ± 679	0.998
Vitamin C (mg/day)	197.2 ± 89.1	217.2 ± 74.8	213.1 ± 92.2	266.6 ± 85.5 *#	0.315
Vitamin D (µg/day)	4.48 ± 2.19	5.69 ± 2.70	5.91 ± 3.77	6.20 ± 4.03	0.300
Vitamin E (mg/day)	11.9 ± 5.4	13.3 ± 2.6	12.0 ± 4.3	13.4 ± 4.9	0.981
Polyphenol intake					
Total polyphenol (mg/day)	593.2 ± 177.4	659.1 ± 184.3	647.6 ± 220.6	735.6 ± 223.7 *	0.571
Flavonoids (mg/day)	367.9 ± 140.1	347.4 ± 105.8	393.6 ± 162.4	447.6 ± 148.1 #	0.131
Phenolic acid (mg/day)	120.7 ± 59.5	171.8 ± 80.6 *	121.4 ± 59.8	163.1 ± 85.5 *	0.852
Lignans (mg/day)	3.56 ± 2.73	15.1 ± 11.1 *	6.08 ± 7.70	8.53 ± 7.60 #	0.002
Stilbens (mg/day)	4.36 ± 3.91	14.7 ± 10.9 *	7.83 ± 7.98	9.00 ± 7.77 #	0.002
Others (mg/day)	84.5 ± 43.0	100.8 ± 44.9	93.4 ± 59.3	92.2 ± 44.2	0.358
Activity					
Sedentary time (hour/day)	7.85 ± 2.13	8.29 ± 1.61	8.03 ± 1.75	7.60 ± 1.82 *	0.082
Total PA (MET·min/week)	3190 ± 2757	3550 ± 2786	2697 ± 1951	3996 ± 3839 *	0.292
Light PA (MET·min/week)	682.1 ± 1122	918.0 ± 878.0	703.4 ± 936.5	1105 ± 1578	0.625
Moderate PA (MET·min/week)	1695 ± 1820	2025 ± 2673	1027 ± 1224	1887 ± 2207	0.586
Vigorous PA (MET·min/week)	812.6 ± 1625	608.0 ± 684.7	910.0 ± 1471	904.3 ± 1827	0.565

Abbreviations: erMedDiet, energy-restricted Mediterranean Diet; MUFA, monounsaturated fatty acid; PUFA, polyunsaturated fatty acid; SFA, saturated fatty acid; trans FA, trans- fatty acid; ω-3 FA, omega-3 fatty acid; ω-6 FA, omega-6 fatty acid; PA, physical activity. Results are presented as mean ± SD (standard deviation). Two-way analysis of co-variance (ANCOVA) after adjustments by age and sex. * Difference in means between participants in time (baseline and 6-year). # Difference in means between groups (responders and non-responders). Data points are significant when *p* < 0.05.

**Table 3:** Oxidative stress biomarkers of MetS participants comparing responders and non-responders at baseline and after the 6-year intervention.

	Non-Responders (*n* = 40)		Responders (*n* = 40)		
	Baseline	6-Year	Baseline	6-Year	*p*-Value
Polyphenols plasma (mg/mL)	0.056 ± 0.019	0.057 ± 0.013	0.057 ± 0.013	0.055 ± 0.008	0.740
Oxidative activities					
CAT (K/L blood)	50.6 ± 19.4	212.4 ± 238.3 *	91.2 ± 156.3	166.4 ± 129.9	0.139
SOD (pkat/L blood)	101.1 ± 94.5	104.9 ± 64.5	103.1 ± 80.2	105.1 ± 58.6	0.906
MPO (μkat/mL blood)	62.1 ± 55.5	55.5 ± 31.4	52.2 ± 28.8	30.6 ± 12.7 *#	0.246
Oxidative damage					
ROS production by neutrophils(RLU/min·10^3^ cells)	2177 ± 1096	1367 ± 614	3151 ± 2014	1519 ± 807 *	0.092
MDA plasma (nM)	1.60 ± 1.58	1.22 ± 0.89	1.28 ± 1.26	0.95 ± 0.79 *	0.499

Abbreviations: CAT, catalase; SOD, superoxide dismutase; MPO, myeloperoxidase; ROS, reactive oxygen species; MDA, malondialdehyde. Results are presented as mean ± SD (standard deviation). Two-way analysis of co-variance (ANCOVA) after adjustments by age and sex. * Difference in means between participants in time (baseline and 6-year). # Difference in means between groups (responders and non-responders). Data points are significant when *p* < 0.05.

## Data Availability

There are restrictions on the availability of data for this trial, due to the signed consent agreements around data sharing, which only allow access to external researchers for studies following the project purposes. Researchers wishing to access the trial data used in this study can make a request to the principal investigator: pep.tur@uib.es.

## References

[1] Rochlani Y., Pothineni N.V., Kovelamudi S., Mehta J.L. (2017). Metabolic syndrome: Pathophysiology, management, and modulation by natural compounds. Ther. Adv. Cardiovasc. Dis..

[2] Masenga S.K., Kabwe L.S., Chakulya M., Kirabo A. (2023). Mechanisms of Oxidative Stress in Metabolic Syndrome. Int. J. Mol. Sci..

[3] Saklayen M.G. (2018). The Global Epidemic of the Metabolic Syndrome. Curr. Hypertens. Rep..

[4] Shin J., Lee J., Lim S., Ha H., Kwon H., Park Y., Lee W., Kang M., Yim H., Yoon K. (2013). Metabolic syndrome as a predictor of type 2 diabetes, and its clinical interpretations and usefulness. J. Diabetes Investig..

[5] Abagre T.A., Bandoh D.A., Addo-Lartey A.A. (2022). Determinants of metabolic syndrome among patients attending diabetes clinics in two sub-urban hospitals: Bono Region, Ghana. BMC Cardiovasc. Disord..

[6] Boudreau D.M., Malone D.C., Raebel M.A., Fishman P.A., Nichols G.A., Feldstein A.C., Boscoe A.N., Ben-Joseph R.H., Magid D.J., Okamoto L.J. (2009). Health Care Utilization and Costs by Metabolic Syndrome Risk Factors. Metab. Syndr. Relat. Disord..

[7] Monserrat-Mesquida M., Quetglas-Llabrés M., Capó X., Bouzas C., Mateos D., Pons A., Tur J.A., Sureda A. (2020). Metabolic Syndrome Is Associated with Oxidative Stress and Proinflammatory State. Antioxidants.

[8] Vona R., Gambardella L., Cittadini C., Straface E., Pietraforte D. (2019). Biomarkers of Oxidative Stress in Metabolic Syndrome and Associated Diseases. Oxid. Med. Cell. Longev..

[9] Sies H. (2015). Oxidative stress: A concept in redox biology and medicine. Redox Biol..

[10] Prieur X., Rőszer T., Ricote M. (2010). Lipotoxicity in macrophages: Evidence from diseases associated with the metabolic syndrome. Biochim. Biophys. Acta Mol. Cell Biol. Lipids.

[11] García-Fontana B., Morales-Santana S., Longobardo V., Reyes-García R., Rozas-Moreno P., García-Salcedo J., Muñoz-Torres M. (2015). Relationship between Proinflammatory and Antioxidant Proteins with the Severity of Cardiovascular Disease in Type 2 Diabetes Mellitus. Int. J. Mol. Sci..

[12] Lastra G., Sowers J.R. (2013). Obesity and cardiovascular disease: Role of adipose tissue, inflammation, and the renin-angiotensin-aldosterone system. Horm. Mol. Biol. Clin. Investig..

[13] Nani A., Murtaza B., Sayed Khan A., Khan N.A., Hichami A. (2021). Antioxidant and Anti-Inflammatory Potential of Polyphenols Contained in Mediterranean Diet in Obesity: Molecular Mechanisms. Molecules.

[14] Gasmi A., Mujawdiya P.K., Noor S., Lysiuk R., Darmohray R., Piscopo S., Lenchyk L., Antonyak H., Dehtiarova K., Shanaida M. (2022). Polyphenols in Metabolic Diseases. Molecules.

[15] de la Rosa L.A., Moreno-Escamilla J.O., Rodrigo-García J., Alvarez-Parrilla E. (2019). Phenolic Compounds. Postharvest Physiology and Biochemistry of Fruits and Vegetables.

[16] Zhang L., Zhang J., Zang H., Yin Z., Guan P., Yu C., Shan A., Feng X. (2024). Dietary pterostilbene exerts potential protective effects by regulating lipid metabolism and enhancing antioxidant capacity on liver in broilers. J. Anim. Physiol. Anim. Nutr..

[17] Guan P., Yu H., Wang S., Sun J., Chai X., Sun X., Qi X., Zhang R., Jiao Y., Li Z. (2024). Dietary rutin alleviated the damage by cold stress on inflammation reaction, tight junction protein and intestinal microbial flora in the mice intestine. J. Nutr. Biochem..

[18] Alberti K.G.M.M., Eckel R.H., Grundy S.M., Zimmet P.Z., Cleeman J.I., Donato K.A., Fruchart J.-C., James W.P.T., Loria C.M., Jr S.C.S. (2009). Harmonizing the metabolic syndrome: A joint interim statement of the International Diabetes Federation Task Force on Epidemiology and Prevention; National Heart, Lung, and Blood Institute; American Heart Association; World Heart Federation; International. Circulation.

[19] Capó X., Ferrer M.D., Olek R.A., Salaberry E., Gomila R.M., Martorell G., Sureda A., Tur J.A., Pons A. (2020). Simultaneous analysis of saturated and unsaturated oxylipins in ‘ex vivo’ cultured peripheral blood mononuclear cells and neutrophils. J. Pharm. Biomed. Anal..

[20] Fernández-Ballart J.D., Piñol J.L., Zazpe I., Corella D., Carrasco P., Toledo E., Perez-Bauer M., Martínez-González M.Á., Salas-Salvadó J., Martn-Moreno J.M. (2010). Relative validity of a semi-quantitative food-frequency questionnaire in an elderly Mediterranean population of Spain. Br. J. Nutr..

[21] Moreiras O., Carbajal A., Cabrera L., Cuadrado C. (2018). Tablas de Composicion de Alimentos (Ciencia Y Tecnica). Guía de Prácticas.

[22] Bouzas C., Bibiloni M.d.M., Julibert A., Ruiz-Canela M., Salas-Salvadó J., Corella D., Zomeño M.D., Romaguera D., Vioque J., Alonso-Gómez Á.M. (2020). Adherence to the Mediterranean Lifestyle and Desired Body Weight Loss in a Mediterranean Adult Population with Overweight: A PREDIMED-Plus Study. Nutrients.

[23] Neveu V., Perez-Jimenez J., Vos F., Crespy V., du Chaffaut L., Mennen L., Knox C., Eisner R., Cruz J., Wishart D. (2010). Phenol-Explorer: An online comprehensive database on polyphenol contents in foods. Database.

[24] Balentine D.A., Dwyer J.T., Erdman J.W., Ferruzzi M.G., Gaine P.C., Harnly J.M., Kwik-Uribe C.L. (2015). Recommendations on reporting requirements for flavonoids in research. Am. J. Clin. Nutr..

[25] Ainsworth B.E., Haskell W.L., Leon A.S., Jacobs D.R., Montoye H.J., Sallis J.F., Paffenbarger R.S. (1993). Compendium of physical activities: Classification of energy costs of human physical activities. Med. Sci. Sports Exerc..

[26] Martínez-González M.A., López-Fontana C., Varo J.J., Sánchez-Villegas A., Martinez J.A. (2005). Validation of the Spanish version of the physical activity questionnaire used in the Nurses’ Health Study and the Health Professionals’ Follow-up Study. Public Health Nutr..

[27] Aebi H. (1984). Catalase in Vitro. Methods Enzymol. Anal..

[28] Flohé L., Ötting F. (1984). Superoxide Dismutase Assay. Methods Enzymol..

[29] Capeillère-Blandin C. (1998). Oxidation of guaiacol by myeloperoxidase: A two-electron-oxidized guaiacol transient species as a mediator of NADPH oxidation. Biochem. J..

[30] Kompaniyets L., Freedman D.S., Belay B., Pierce S.L., Kraus E.M., Blanck H.M., Goodman A.B. (2023). Probability of 5% or Greater Weight Loss or BMI Reduction to Healthy Weight among Adults with Overweight or Obesity. JAMA Netw. Open.

[31] Hall K.D., Kahan S. (2018). Maintenance of Lost Weight and Long-Term Management of Obesity. Med. Clin. N. Am..

[32] Rothberg A.E., McEwen L.N., Kraftson A.T., Ajluni N., Fowler C.E., Nay C.K., Miller N.M., Burant C.F., Herman W.H. (2017). Impact of weight loss on waist circumference and the components of the metabolic syndrome. BMJ Open Diabetes Res. Care.

[33] Zhang X., Wang Y., Li Y., Gui J., Mei Y., Yang X., Liu H., Guo L., Li J., Lei Y. (2024). Four-years change of BMI and waist circumference are associated with metabolic syndrome in middle-aged and elderly Chinese. Sci. Rep..

[34] Yang S., Zhou Z., Miao H., Zhang Y. (2023). Effect of weight loss on blood pressure changes in overweight patients: A systematic review and meta-analysis. J. Clin. Hypertens..

[35] Ryder E., Diez-Ewald M., Mosquera J., Fernández E., Pedreañez A., Vargas R., Peña C., Fernández N. (2014). Association of obesity with leukocyte count in obese individuals without metabolic syndrome. Diabetes Metab. Syndr. Clin. Res. Rev..

[36] Tussing-Humphreys L., Lamar M., McLeod A., Schiffer L., Blumstein L., Dakers R., Karstens A., Hemphill N.O.N., Strahan D., Siegel L. (2022). Effect of Mediterranean diet and Mediterranean diet plus calorie restriction on cognition, lifestyle, and cardiometabolic health: A randomized clinical trial. Prev. Med. Rep..

[37] Lotfi K., Saneei P., Hajhashemy Z., Esmaillzadeh A. (2022). Adherence to the Mediterranean Diet, Five-Year Weight Change, and Risk of Overweight and Obesity: A Systematic Review and Dose–Response Meta-Analysis of Prospective Cohort Studies. Adv. Nutr..

[38] van Baak M.A. (2021). Dietary carbohydrates and weight loss maintenance. Curr. Opin. Clin. Nutr. Metab. Care.

[39] Hardy D.S., Garvin J.T., Xu H. (2020). Carbohydrate quality, glycemic index, glycemic load and cardiometabolic risks in the US, Europe and Asia: A dose–response meta-analysis. Nutr. Metab. Cardiovasc. Dis..

[40] Velázquez-López L., Santiago-Díaz G., Nava-Hernández J., Muñoz-Torres A.V., Medina-Bravo P., Torres-Tamayo M. (2014). Mediterranean-style diet reduces metabolic syndrome components in obese children and adolescents with obesity. BMC Pediatr..

[41] Wong S.K., Chin K.-Y., Ima-Nirwana S. (2020). Vitamin C: A Review on its Role in the Management of Metabolic Syndrome. Int. J. Med. Sci..

[42] Ramaiah P., Baljon K.J., Hjazi A., Qasim M.T., Salih Al-ani O.A., Imad S., Hussien B.M., Alsalamy A., Garousi N. (2024). Dietary polyphenols and the risk of metabolic syndrome: A systematic review and meta-analysis. BMC Endocr. Disord..

[43] Wan Y., Ma D., Shang Q., Xu H. (2024). Association between dietary flavonoid intake and hypertension among U.S. adults. Front. Immunol..

[44] Gouveia H.J.C.B., Urquiza-Martínez M.V., Manhães-de-Castro R., Costa-de-Santana B.J.R., Villarreal J.P., Mercado-Camargo R., Torner L., de Souza Aquino J., Toscano A.E., Guzmán-Quevedo O. (2022). Effects of the Treatment with Flavonoids on Metabolic Syndrome Components in Humans: A Systematic Review Focusing on Mechanisms of Action. Int. J. Mol. Sci..

[45] Guo X., Tresserra-Rimbau A., Estruch R., Martínez-González M., Medina-Remón A., Fitó M., Corella D., Salas-Salvadó J., Portillo M., Moreno J. (2017). Polyphenol Levels Are Inversely Correlated with Body Weight and Obesity in an Elderly Population after 5 Years of Follow Up (The Randomised PREDIMED Study). Nutrients.

[46] Esposito K., Kastorini C.-M., Panagiotakos D.B., Giugliano D. (2011). Mediterranean Diet and Weight Loss: Meta-Analysis of Randomized Controlled Trials. Metab. Syndr. Relat. Disord..

[47] Franz M.J., Boucher J.L., Rutten-Ramos S., VanWormer J.J. (2015). Lifestyle Weight-Loss Intervention Outcomes in Overweight and Obese Adults with Type 2 Diabetes: A Systematic Review and Meta-Analysis of Randomized Clinical Trials. J. Acad. Nutr. Diet..

[48] Myers J., Kokkinos P., Nyelin E. (2019). Physical Activity, Cardiorespiratory Fitness, and the Metabolic Syndrome. Nutrients.

[49] Adenan D.M., Jaafar Z., Jayapalan J.J., Abdul Aziz A. (2020). Plasma antioxidants and oxidative stress status in obese women: Correlation with cardiopulmonary response. PeerJ.

[50] Tumova E., Sun W., Jones P.H., Vrablik M., Ballantyne C.M., Hoogeveen R.C. (2013). The Impact of Rapid Weight Loss on Oxidative Stress Markers and the Expression of the Metabolic Syndrome in Obese Individuals. J. Obes..

[51] Klebanoff S.J. (2005). Myeloperoxidase: Friend and foe. J. Leukoc. Biol..

[52] Chen S.-J., Yen C.-H., Huang Y.-C., Lee B.-J., Hsia S., Lin P.-T. (2012). Relationships between Inflammation, Adiponectin, and Oxidative Stress in Metabolic Syndrome. PLoS ONE.

[53] Monserrat-Mesquida M., Quetglas-Llabrés M., Bouzas C., García S., Mateos D., Gómez C., Gámez J.M., Poulsen H.E., Tur J.A., Sureda A. (2022). Effects of 2-Year Nutritional and Lifestyle Intervention on Oxidative and Inflammatory Statuses in Individuals of 55 Years of Age and over at High Cardiovascular Risk. Antioxidants.

[54] Crujeiras A.B., Parra D., Abete I., Martínez J.A. (2007). A hypocaloric diet enriched in legumes specifically mitigates lipid peroxidation in obese subjects. Free Radic. Res..

[55] Furukawa S., Fujita T., Shimabukuro M., Iwaki M., Yamada Y., Nakajima Y., Nakayama O., Makishima M., Matsuda M., Shimomura I. (2004). Increased oxidative stress in obesity and its impact on metabolic syndrome. J. Clin. Investig..

[56] Fraga C.G., Celep G.S., Galleano M. (2009). Biochemical actions of plant phenolics compounds: Thermodynamic and kinetic aspects. Plant Phenolics Hum. Health Biochem. Nutr. Pharmacol..

[57] Moschen A.R., Molnar C., Enrich B., Geiger S., Ebenbichler C.F., Tilg H. (2011). Adipose and Liver Expression of Interleukin (IL)-1 Family Members in Morbid Obesity and Effects of Weight Loss. Mol. Med..

[58] Kanda H. (2006). MCP-1 contributes to macrophage infiltration into adipose tissue, insulin resistance, and hepatic steatosis in obesity. J. Clin. Investig..

[59] Fu C.-P., Sheu W.H.-H., Lee I.-T., Lee W.-J., Wang J.-S., Liang K.-W., Lee W.-L., Lin S.-Y. (2015). Weight loss reduces serum monocyte chemoattractant protein-1 concentrations in association with improvements in renal injury in obese men with metabolic syndrome. Clin. Chem. Lab. Med..

[60] Jung S.H., Park H.S., Kim K.-S., Choi W.H., Ahn C.W., Kim B.T., Kim S.M., Lee S.Y., Ahn S.M., Kim Y.K. (2008). Effect of weight loss on some serum cytokines in human obesity: Increase in IL-10 after weight loss. J. Nutr. Biochem..

[61] Monserrat-Mesquida M., Quetglas-Llabrés M., Bouzas C., Capó X., Mateos D., Ugarriza L., Tur J.A., Sureda A. (2021). Peripheral blood mononuclear cells oxidative stress and plasma inflammatory biomarkers in adults with normal weight, overweight and obesity. Antioxidants.

